# 4301 Racial/Ethnic variations in discharge destination after inpatient care: A risk-adjusted analysis of a large regional dataset

**DOI:** 10.1017/cts.2020.278

**Published:** 2020-07-29

**Authors:** Arnab Kumar Ghosh, Said Ibrahim

**Affiliations:** 1 Weill Cornell Medical College of Cornell University

## Abstract

OBJECTIVES/GOALS: While there are many well-documented factors for racial/ethnic variation in discharge destination, less is known about the role hospital processes play. We hypothesize that variation in hospital processes -defined as the patient length of stay (LOS) adjusted for known confounders - explains racial/ethnic variation in discharge destination. METHODS/STUDY POPULATION: Our sample was 176,686 discharges from 165 hospitals in 2014 using the New York State Inpatient Dataset from the Healthcare Cost and Utilization Project, merged with the 2014 American Hospital Association Annual Survey to build a file of inpatient discharges with patient, disease and socio-economic characteristics. We excluded patients under 18 years, those with LOS of zero, those who died, those admitted to critical access hospitals, and patients from hospitals that lacked sufficient number of minority patients. We used a generalized linear mixed effects model to create an in-hospital risk-adjusted LOS by modelling the relationship between the interaction of race and discharge destination and LOS, controlling for known confounders such as patient, disease and between-hospital characteristics. RESULTS/ANTICIPATED RESULTS: Mean age of sample was 56.5 years, 57.3 % female, 54.9% white, 18.9% black and 13.1% Hispanic; 64.3% were discharged home, 15.8% to a skilled nursing or other intermediate care facility, 15.5% to home with home care and 2.4% left against medical advice. The top 3 discharge diagnoses were vaginal delivery (6.3% of discharges), psychosis (4.7%), and major joint replacement (2.9%). In adjusted analysis compared to white patients, black and Hispanic patients did not have an risk of increased LOS after being discharged to non-home destinations vs. discharged home, (black patients, adjusted OR [AOR], 0.97; 95% CI: 0.94-1.00, p = 0.08; Hispanic patients, AOR, 1.01; 95% CI: 0.98 – 1.05, p = 0.5). However, being black compared to white and discharge to non-home destinations significantly increased LOS. DISCUSSION/SIGNIFICANCE OF IMPACT: In this large sample of patients admitted for inpatient care in 2014 in New York, we found no independent effect between race and discharge destination on a patient’s LOS after controlling for patient, disease and between-hospital characteristics. However race/ethnicity increased LOS, suggesting its effect may play a role on in-hospital processes CONFLICT OF INTEREST DESCRIPTION: Dr. Ghosh has no relevant relationships with commercial interests to disclose Dr. Ibrahim has no relevant relationships with commercial interests to disclose

